# Degradation of Contaminants of Emerging Concern by Electrochemical Oxidation: Coupling of Ultraviolet and Ultrasound Radiations

**DOI:** 10.3390/ma13235551

**Published:** 2020-12-05

**Authors:** María J. Martín de Vidales, Jaime Rua, José Luis Montero de Juan, Francisco Fernández-Martínez, Antonio J. Dos santos-García

**Affiliations:** Mechanical, Chemical and Industrial Design Engineering Department, ETSIDI, Universidad Politécnica de Madrid (UPM), Ronda de Valencia 3, 28012 Madrid, Spain; mariajose.martindevidales@upm.es (M.J.M.d.V.); jaimerua94@hotmail.com (J.R.); jl.montero@upm.es (J.L.M.d.J.); Francisco.fernandezm@upm.es (F.F.-M.)

**Keywords:** wastewater treatment, electrochemical oxidation, contaminants of emerging concern, photo-electrochemical oxidation, sono-electrochemical oxidation, photocatalysis

## Abstract

In this work, we study the electrochemical oxidation of methyl red, a dye present in textile industrial effluents, which is selected as the model for the degradation of Contaminants of Emerging Concern. The influence of the initial pollutant concentration (1–5 mg dm^−3^), applied current density (2–15 mA cm^−2^), and the coupling of ultraviolet or ultrasound radiation have been studied using a titanium plate as anode. The results show that electrochemical oxidation is able to efficiently remove methyl red, and the process efficiency decreases with the initial pollutant concentration. At high applied current densities, efficiency drastically decreases due to a less effective mass transfer of the pollutant on the anodic surface. On one hand, the coupling of ultrasound entails an antagonistic effect on the process efficiency, which is probably due to a massive formation of oxidant radicals followed by a fast recombination process. On the other hand, the coupling of ultraviolet radiation increases the process efficiency. Concomitantly to the oxidation processes, titanium electrode produces rising TiO_2_–anatase nanoparticles, boosting the mineralization process. This new finding sets up a significant improvement over conventional photocatalysis treatments using TiO_2_–anatase as a catalyst due to synergistic effects coming from the coupling of the electrochemical oxidation and photocatalysis process with Ti anode.

## 1. Introduction

In the last few decades, the scientific community has raised awareness because of the continuous discharge of explicit compounds to the environment and its persistent presence in the medium. These are the so-called Contaminants of Emerging Concern (CECs), which are hard-to-degrade by conventional treatments, and their presence in water and wastewater can lead to unpredictable consequences in aquatic ecosystems or even in whole living beings [1]. There are an increasing number of research studies concerning the effects in human health. For instance, researchers have reported damages in the endocrine, immune, and neurological systems, and established some relationships between CECs and hormonal control or the diagnosis of several types of cancer (breast, ovary, prostate, testes, etc.) [2].

In light of these findings, new approaches are needed, taking into account that conventional treatments performed in Municipal Wastewater Treatment Plants (MWTP) cannot completely remove those pollutants. Nowadays, Electrochemical Oxidation (EO) is considered, among other Advanced Oxidation Process (AOP), as a promising technique to remove CECs from water and wastewater [3,4,5]. However, there are still some drawbacks, related to mass transfer, that have to be overcome. Thus, the higher the organic concentration, the higher the efficiency achieved. In order to improve the process efficiency, an ultrasound generator can be inserted into the system, since it is able to improve the mass transfer and the electrohydraulic cavitation, promoting the production of hydroxyl radicals through water decomposition. Furthermore, the coupling of EO with ultrasound Sono-EO entails the electrohydraulic cavitations, growth, and cyclical collapse of gas bubbles [6,7,8,9].

Ultraviolet (UV) light radiation can be coupled with the electrochemical process (Photo-EO) in order to increase the generation and/or activation of oxidizing agents in the bulk [10,11,12,13,14,15]. The coupling of UV radiation can lead to a synergistic effect explained either by heterogeneous and/or homogenous catalytic processes [16,17,18]. From one hand, in a heterogeneous catalytic process, the external bias contributes to a decreased electron–hole pair recombination process at the electrode surface, boosting the generation of excited radicals. On the other hand, the electrochemically generated species are susceptible to be photo activated through a homogeneous catalytic process.

In the recent years, conventional electrochemical oxidation and the coupling of irradiated techniques to electrochemical oxidation have been applied to the treatment of wastewater polluted with different organic pollutants [19,20,21]. However, this coupling has been studied with typical and commercial anodes, such as DSA (dimensionally stable anode), BDD (boron doped diamond), or platinum. In this work, the electrochemical oxidation will be studied with titanium anode, looking for the formation of TiO_2_ nanoparticles through a further oxidation of the anode, including thereby a photocatalysis process to the medium.

Methyl red (2-(N,N-dimethyl-4-aminophenyl) azobenzenecarboxylic acid is a member of azobenzenes consisting of benzoic acid substituted at position 2 by a 4-[(dimethylamino)phenyl]diazenyl group. It is extensively used as a dye in the textile industry and its massive presence in the discharge of effluents from these industries makes methyl red (MR) a pollutant that has to be considered as CEC. Furthermore, there is no current legislation from different countries for the regulation of its introduction into the environment in a persistent way or the effects affecting the surface, ground, and drinking water [22,23,24,25].

Taking into account all these considerations, the goal of this work is the evaluation of EO as potential technique for the degradation CEC from wastewater. Operation conditions such as the initial concentration of MR as a target pollutant (1–5 mg dm^−3^) and applied current density (2–15 mA dm^−3^) are evaluated. All the studies were conducted on MR aqueous solutions electrolyzed with a titanium anode. The removal of this CEC was studied with the coupling of EO with ultrasound (Sono-EO) or UV radiation (Photo-EO). A comparison between sonolysis, photolysis, EO, Sono-EO, and Photo-EO was made in order to find the optimal technology for the degradation of CECs.

## 2. Materials and Methods

### 2.1. Chemicals

Methyl red (C_15_H_15_N_3_O_2_), Ti plates (99.9% purity) and sodium chloride (>99.0% purity) were supplied by Sigma-Aldrich (Steinheim, Germany). Sulfuric acid and sodium hydroxide were used to adjust the pH of the cleaning solution, and they were of analytical grade and supplied by Panreac Química S.A. (Barcelona, Spain).

### 2.2. Analytical Procedures

Measurements of pH were carried out with an InoLab WTW pH-meter.

The evolution of MR concentration during the experiments was monitored by UV spectrophotometry in a UVIKON 941 plus analyzer (Kontron Instruments, Watford, Herts, UK) at a wavelength λ_MR_ = 423 nm. A calibration plot was constructed by measuring the absorbance of MR in five standard solutions, obtaining the following relation: C_MR_ = 20.941·Abs − 1.0536 (Figure of merit: R^2^ = 0.9992).

X-ray powder diffraction (XRD) of TiO_2_ nanoparticles was performed in a D5000 diffractometer (Siemens/Bruker, Karlsruhe, Germany) using Cu K_α1,2_ radiation operating under 30 mA and 40 kV.

### 2.3. Experimental Procedures

Bench-scale electrochemical oxidation treatments were conducted under batch operation mode. The initial concentration of the pollutant was 1–5 mg dm^−3^ (aqueous solution of MR), covering both the range of a polluted industrial waste or a stockpile [26] and small concentrations found in effluents of a Municipal Wastewater Treatment Plant [27]. In addition, NaCl (0.051 M) was used as supporting electrolyte for the electrochemical processes. Solution was treated in a borosilicate open reactor (600 mL) with a heating/cooler jacket keeping the temperature constant at 25 °C and under continuous stirring (50% power of a conventional laboratory stirrer) during 120 min. Square titanium plates (50 mm × 50 mm) were used as anode and cathode (99.9% purity). Both electrodes were submerged in the solution with an electrode gap of 10 mm. Filtered aliquots of 10 mL were taken for analysis during the experiments.

The electrodes were polarized for 10 min at 30 mA cm^−2^, before galvanostatic electrolysis assays, using a cleaning solution of 0.035 M Na_2_SO_4_ at pH = 2, to remove impurities from the electrode surface. The cell voltage was keeping constant (≈18 V) during electrolysis, indicating that anode layers did not undergo appreciable deterioration or passivation phenomena.

**Figure 1:**
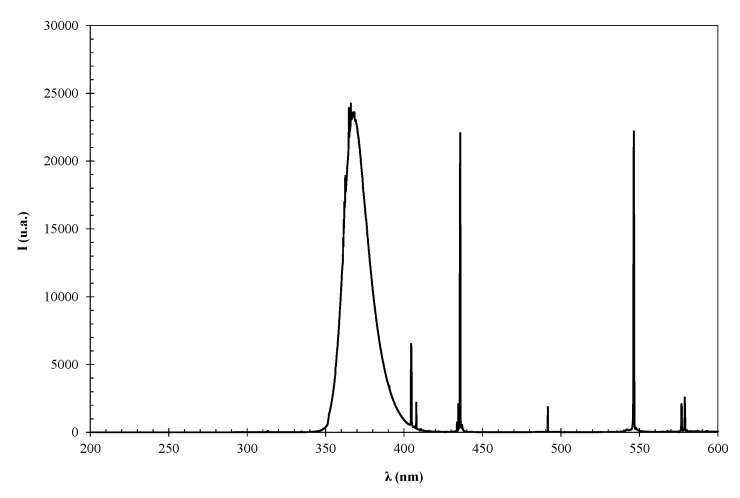
UV lamp spectrum.

The UV source used in the photolysis, Photo-EO, and photocatalysis processes was a Phillips Actinic B.L. lamp (Philips lighting, Madrid, Spain) with two Hg UV light bulbs 8 W power and predominant UV-A radiation, as Figure 1 shows). The lamp was placed on the solution at the minimum distance (5.5 cm) located between the electrodes in order to improve the efficiency of the process.

For sonolysis and Sono-EO processes, the reactor was placed in an ultrasound bath 40 kHz (low frequency, J. P. Selecta, Barcelona, Spain), 100 W [15,18].

## 3. Results and Discussion

### 3.1. Electrochemical Oxidation

**Figure 2:**
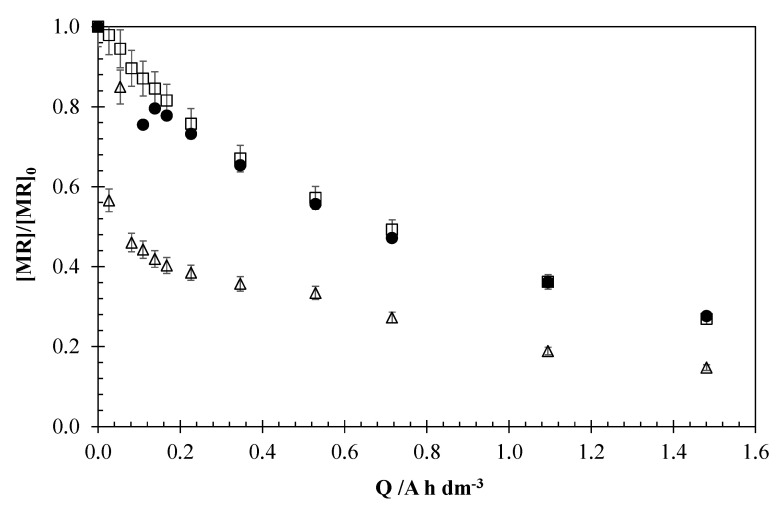
Influence of the initial concentration of the pollutant on the Electrochemical Oxidation (EO) of methyl red (MR). Operation conditions: 15 mA cm^−2^, 0.051 M NaCl. (□) 5 mg dm^−3^; (●) 2.5 mg dm^−3^; (Δ) 1 mg dm^−3^.

Figure 2 shows the evolution of the normalized concentration of MR against applied electric charge for different initial concentration of the pollutant on the solution (5, 2.5, and 1 mg dm^−3^). The applied current density was 15 mA cm^−2^, and the concentration of the supporting NaCl electrolyte was 0.051 M. This electrolyte was used to promote the formation of ClO^−^ as an oxidizing agent.

A high rate MR degradation was observed in all cases. The samples containing an initial concentration ranging between 2.5 and 5 mg dm^−3^ reached a final degradation of 70%, while a degradation of 85% was found by decreasing the initial concentration down to 1 mg dm^−3^. This phenomenon can be explained based on the mineralization of MR by the oxidative action of the hydroxyl radicals (•OH), which are electrogenerated in the anodic surface by the oxidative decomposition of water. For instance, the generation of other oxidizing agent such as hypochlorite, ozone, or hydrogen peroxide can also take place, but in a minor extension [28,29]. The process efficiency decreases with the initial concentration of the pollutant, mainly between 2.5 and 1 mg dm^−3^. It can be explained in light of the existence of indirect oxidation by the electrogenerated oxidants, following a pseudo-first order kinetic [30,31], which depends both on the concentration of pollutant and of the oxidizing species, including the last one in the apparent kinetic constant Equation (1).

r = K·[MR] = k’·[Oxidizing agent]·[MR] (1)

In steady state, the difference between the electrolytically produced oxidants and those spent in the oxidation of organics (pseudo-stationary concentration) decreases with the concentration of the pollutant that can be oxidized. This causes a decrease of the term k’·[Oxidizing agent] in Equation (1) and, consequently, in the process efficiency.

The degradation profile is rather similar in the three set of experiments. Initially, for electric charges lower than 0.4 A h dm^−3^, a fast pollutant decrease can be observed, but for higher values, the degradation is progressively slower. This behavior can be explained on the basis that in the initial moments of the experiment, the oxidation of MR depends on the production and action of the hydroxyl radicals and other oxidizing agents. As the process continues, the MR concentration decreases and the control by mass transfer of the organic pollutant to the anodic surface gains importance, causing a decrease of the degradation rate and the process efficiency.

As it has been stated before, NaCl was used as supporting electrolyte in order to increase the ionic conductivity in the system. Thus, the presence of the chloride anion in the solution leads to its oxidation to chlorine gas in Equations (2) and (3) by its electrochemical oxidation [30]. When followed by the chemical disproportionation of chlorine (reduction-oxidation, Equation (4)), this entails the generation of hypochlorite, which can be oxidized to different oxoanions of chlorine: chlorite, chlorate, and perchlorate according to Equations (5)–(7).

Cl^−^ → •Cl + e^−^ (2)

2 •Cl ↔ Cl_2_ (3)

Cl_2_ + H_2_O → Cl^−^ + ClO^−^ + 2 H^+^ (4)

ClO^−^ + H_2_O → ClO_2_^−^ + 2H^+^ + 2e^−^ (5)

ClO_2_^−^ + H_2_O → ClO_3_^−^ + 2H^+^ + 2e^−^ (6)

ClO_3_^−^ + H_2_O → ClO_4_^−^ + 2H^+^ + 2e^−^ (7)

The hypochlorite ion, with high oxidizing power, has been extensively used in the oxidation of micropollutants. However, there are still several drawbacks that have to be addressed, since the presence of chlorate and perchlorate in the aquatic medium entails a high toxicity [31]. Furthermore, if the concentration of these species is high, other techniques should be applied to eliminate them after the treatment, such as adsorption with activated carbon or H_2_O_2_ addition [32,33].

**Figure 3:**
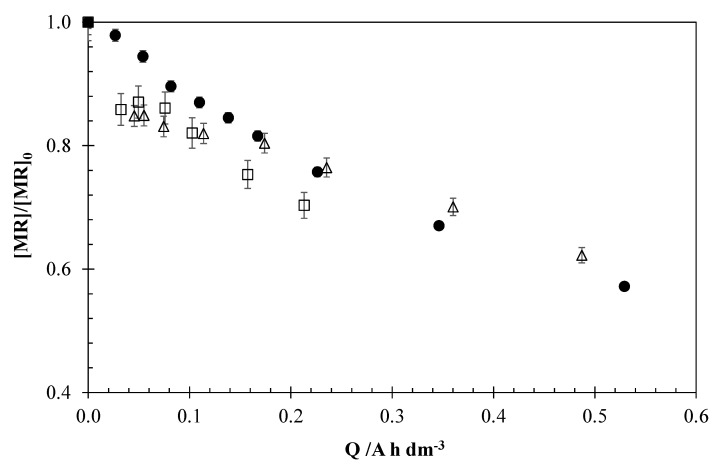
Influence of the applied current density on the EO of MR. Operation conditions: [MR]_0_ = 5 mg dm^−3^, 0.051 M NaCl. (□) 2 mA cm^−2^, (Δ) 5 mA cm^−2^, (●) 15 mA cm^−2^.

Figure 3 shows the profiles of normalized MR concentrations for different applied current densities (2, 5, and 15 mA cm^−2^), keeping constant the initial concentration of the pollutant (5 mg dm^−3^).

This figure shows that although there are small differences, an increase of the applied current density leads to a decrease of the process efficiency, since larger charges are required to obtain the same removal. This demonstrates that the process is controlled by the mass transfer of the pollutant to the anodic surface, where the vast majority of the electrogenerated oxidants are found. In this sense, the differences observed between the experiments are higher when there are less organic compounds in the medium and the control by the mass transfer is more important. This behavior can be observed mainly between 2 and 5 mA cm^−2^, where the efficiency achieved is similar in the first part of the tests, but for values of electric charge higher than 0.15 A h dm^−3^, the experiment carried out with a current density of 5 mA cm^−2^ presents a similar efficiency to the observed applying 15 mA cm^−2^.

It is important to highlight that a white solid was formed during the degradation process, which was probably due to damages caused in the electrodes. Therefore, this solid should correspond to the formation of TiO_2_ nanoparticles coming from the oxidation of the titanium plate used as anode. In order to shed light into this observation, XRD analysis of the solid was performed.

**Figure 4:**
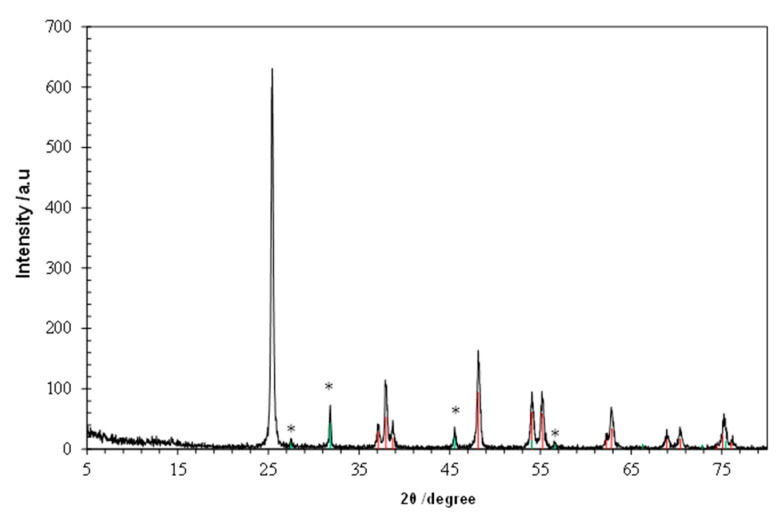
XRD pattern of the generated solid. Maxima noted with an asterisk belong to NaCl.

Figure 4 shows the X-ray diffraction pattern of the obtained solid. The main diffraction maxima can be indexed with a tetragonal *I*4_1_/*amd* space group, corresponding to the characteristic diffraction peaks of anatase (TiO_2_) [34]. It is also worth mentioning that other diffraction maxima also appear corresponding to NaCl, coming from the electrolyte [35].

### 3.2. Coupling of Irradiated Techniques

In order to look for the improvement of the process efficiency of the electrochemical oxidation of MR, ultrasound or ultraviolet radiations were coupled to the system.

**Figure 5:**
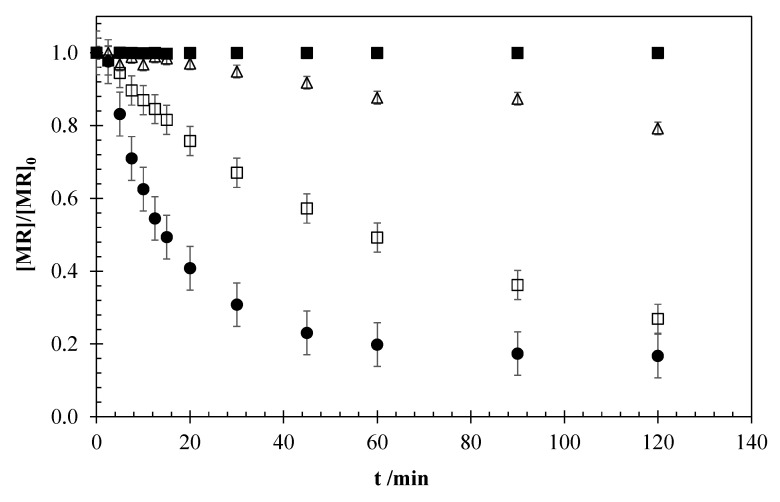
MR concentration profiles for the experiments conducted by photolysis (■), EO (□), photo-electrochemical oxidation (Photo-EO) (●), and photocatalysis (Δ). Operation conditions: [MR]_0_ = 5 mg dm^−3^, 15 mA cm^−2^, 0.051 M NaCl, UV-A 8 W.

Figure 5 shows the comparison between the experiments carried out by electrochemical oxidation (EO) and photo-electrochemical oxidation (Photo-EO) with the same operation conditions and the coupling of UV radiation. For a better comparison, a test of photolysis was also carried out. It is important to highlight that in the Photo-EO process, the presence of TiO_2_ anatase nanoparticles in the medium coming from anode degradation can entail a photocatalytic process. In this sense, a test of photocatalysis with dispersed TiO_2_ anatase (commercial compound) was conducted, with the same g/L load of TiO_2_ than that generated on the EO experiment at 15 mA cm^−2^ (the higher current density, the higher generation of TiO_2_).

It can be observed that photolysis and photocatalysis with dispersed TiO_2_ only reach 0.5% and 20% removal, respectively, in two hours. However, the coupling of UV radiation to the EO system (Photo-EO) entails a high increase of the process efficiency, reaching 80% removal in only 50 min, versus the 52% obtained by EO. This behavior can be based on a higher generation of oxidizing agents and/or the promotion of the activation of oxidants produced electrochemically to the formation of radicals [13,14,18]. The anodic production of hydroxyl radicals in EO [36] can be enhanced via Equations (8) and (9), which is caused by the conversion to other oxidizing species (H_2_O_2_ and O_3_) under UV radiation. In addition, Cl^−^ can be activated to form ●Cl radical Equation (10). In this case, in contrast to the results observed for a single electrolytic process, the effect of these radicals can be extended to the bulk phase, significantly improving the removal efficiency [15,18].

H_2_O_2_ → 2 •OH (8)

H_2_O_2_ + 2 O_3_→ 2 •OH + 3 O_2_ (9)

Cl^−^ + hυ→ •Cl (10)

An incorporation of the photocatalytic process with TiO_2_ anatase (active catalytic phase [37]) is possible due to the generation of this semiconductor by the oxidation of the titanium plate used as anode.

Finally, both contributions are possible.

A comparison between the photocatalysis, EO, and Photo-EO processes allows us to confirm that both contributions are taking place in the Photo-EO experiment, and this entails a synergistic effect in the system with a high process efficiency for the removal of MR. In addition, the efficiency of TiO_2_ can change when it is suspended in the aqueous medium and when it is attached to the electrode surface. In this sense, it is demonstrated that the efficiency increases when TiO_2_ is formed by the oxidation of the Ti electrode, and the photocatalysis processes can be coupled to the electrochemical oxidation technology. This is a very important result, because it demonstrates that the coupling of EO with a sacrificial titanium anode and UV radiation can be a promising technology for the treatment of wastewater polluted with CECs, obtaining a high pollutant removal at low reaction time. The use of titanium plates as anode and cathode entails the possibility of reversing the potential of the power supply so that the sacrificial anode acts as a cathode and vice versa. In this way, the deterioration of the electrodes is shared.

**Figure 6:**
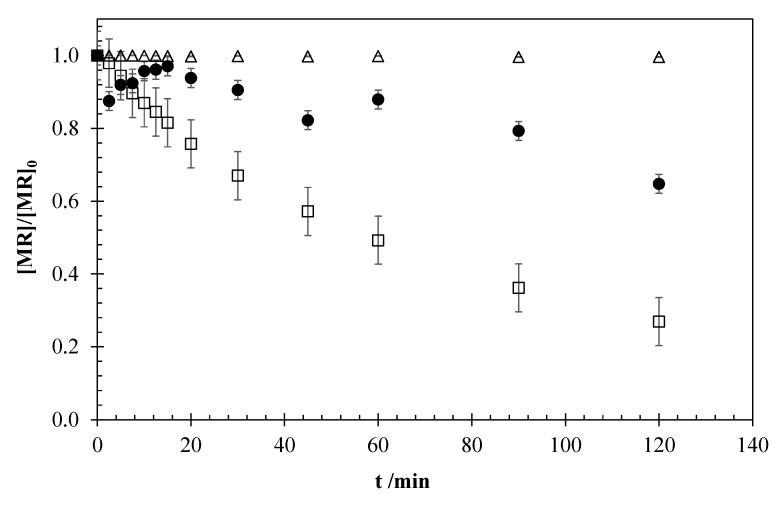
MR concentration profiles obtained for the experiments of sonolysis (Δ), EO (□), and the coupling of EO with ultrasound (Sono-EO) (●). Operation conditions: [MR]_0_ = 5 mg dm^−3^, 15 mA cm^−2^, 0.051 M NaCl, US-40 kHz 100 W.

Figure 6 shows the influence of the coupling of ultrasound radiation (40 kHz, 100 W) to the EO system. A sonolysis test was also carried out in order to evaluate the radiation contribution.

As it can be observed, the sonolysis process achieves a negligible degradation of the pollutant (0.4%). When the ultrasound bath is coupled to the system, the process efficiency decreases, and an antagonistic effect is observed. As commented before, irradiated systems favor the generation and activation of oxidants via the formation of radical species. Therefore, the observation of an antagonistic effect in the system with the US radiation coupling may be due to a massive formation of oxidizing agents that would combine with each other to form species with greater chemical stability and lower oxidation capacity, preventing their direct or indirect action for the oxidation of MR Equations (11) and (12) [18].

2 •OH→ H_2_O_2_ (11)

H_2_O_2_→ H_2_O +1/2 O_2_ (12)

Thus, this negative effect can counteract the possibility of decreasing mass transfer control by US radiation, promoting the decomposition of water that produces hydroxyl radicals and electrohydraulic cavitation, growth, and the cyclical collapse of gas bubbles [6,7,8,9].

### 3.3. Kinetic, Efficiency, and Energy Consumptions

The representation of the data in semi-logarithmic scale follows a linear trend that, at first sight, suggest a first-order kinetic with respect to the concentration of MR. It can be easily explained in terms of a mass transfer control of the process, taking into account the small concentration of the pollutant, which is many times below the limit concentration at the applied current density. However, the kinetic constant, represented by the slope of this linear trend, depends on the particular electrolysis and, consequently, on the initial concentration of MR, being smaller at larger concentration, as can be observed in Table 1.

As it was commented before, it can be due to the existence of indirect oxidation mechanisms with electro-generated oxidants and a second-order kinetic model k’·[Oxidizing agent]·[Organic]. At this point, it is well known that other oxidant species are produced during the electrochemical process. Among them, persulfates, ozone, or hydrogen peroxide but also powerful radicals such as the hydroxyl radical. The pseudo-stationary concentration of these oxidants (difference at the steady-state between the oxidant produced electrolytically and the oxidant spent in the oxidation of organics) concomitantly decreases with the concentration of organic compounds, producing the decrease of the k’·[Oxidizing agent] term, behaving therefore as a pseudo-first-order kinetic constant.

Table 1 also shows that regarding the applied current density, it is observed that the kinetic constant increases with the current density. This parameter is directly related to the generation of oxidizing species on the anode surface [38], and a higher concentration of oxidizing species causes an increase in the apparent kinetic constant and, therefore, a higher degradation rate of degradation of RM in the medium.

In the Sono-EO and Photo-EO tests, the kinetic constant decreases compared to the electrochemical oxidation test. This phenomenon can be explained based on the combination of different advanced oxidation processes that can produce the recombination of excessively generated radicals that generates an antagonistic effect that reduces the apparent kinetic constant.

Between the Photo-EO and photocatalysis tests, there is no significant variation in the kinetic constant. However, in the Sono-EO test, a kinetic constant is obtained approximately two times higher than in the Photo-EO test. This improvement may be due to the use of ultrasound radiation to improve the mass transfer to the anodic surface [39].

In addition, it is observed that the efficiency decreases with the initial concentration of the pollutant, having a significant change in the test of 1 mg dm^−3^, as was observed in the study of the kinetic, since in these experiments, the current density remains constant and, therefore, the resources used do as well.

On the other hand, the efficiency of the process increases with the decrease of the applied current density. This behavior is typical for systems controlled by the mass transfer, since all the energy resources are not properly used when the applied current density is increased.

In the Sono-EO and Photo-EO tests, the efficiency obtained has a fairly low value, which shows, once again, the antagonistic effect observed with the coupling of UV or US radiation to the electrochemical oxidation system.

If energy consumptions are calculated, it is observed that in the EO, Sono-EO, and Photo-EO processes with an applied current density of 15 mA cm^−2^, 23.98, 441.02, and 60.06 kW h m^−3^ are consumed, respectively. Thus, in this context, and due to the efficiency of EO process being higher, this is the most recommendable technology for the removal of MR from wastewater.

## 4. Conclusions

This study demonstrates that electrochemical oxidation with titanium anode allows degrading methyl red, which is chosen as model of CEC, efficiently (70% removal with an applied electric charge of 1.5 A h dm^−3^ from a solution polluted with 5 mg dm^−3^ of MR at 15 mA cm^−2^).

The process efficiency decreases with the applied current density, although this influence is not significant. Thus, the process is controlled by the mass transfer of the pollutant to the anodic surface, where most of the electrogenerated oxidizing species can be found.

Sonolysis and photolysis techniques were studied, and it was observed that they only entail a slight degradation of the studied pollutant. However, the process efficiency can be improved with the coupling of UV radiation to the EO system (Photo-EO) increasing the formation of oxidant agents (80% removal versus 52%, in 50 min). This improvement can be also due to the photocatalysis contribution with the formation of TiO_2_ anatase by the oxidation of the titanium plate used as anode. The formation of anatase was confirmed by XRD analysis. Thus, this phenomenon opens the doors to a new application of electrochemical oxidation with a Ti plate used as a sacrificial anode, because it can entail a synergistic effect with the coupling of electrochemical oxidation and the photocatalysis process.

Finally, an antagonistic effect is observed with the coupling of UV radiation to the EO system. This can be due to an excessive formation of oxidants that, instead of oxidizing organic pollutants, recombine with each other to form more stable species with less oxidative capacity.

## Figures and Tables

**Table 1:** Kinetic constant and efficiency calculated for the different experiments conducted.

Process	[MR]_0_ (mg dm^−3^)	Current Density (mA cm^−2^)	Kinetic Constant (min^−1^)	Efficiency (dm^3^ A^−1^ h^−1^)
EO	5	15	0.0135	1.19
EO	2.5	15	0.0152	1.34
EO	1	15	0.0817	7.44
EO	5	5	0.0084	1.69
EO	5	2	0.0034	2.39
Sono-EO	5	15	0.0032	0.28
Photo-EO	5	15	0.0018	0.16
Photocatalysis	5	-	0.0016	-
